# The Object of Exercise

**Published:** 1883-11

**Authors:** 


### THE OBJECT OF EXERCISE.

IF a strong active man, in perfect health, were to lie down in idleness during a few months, his strength and vigor would depart from him, his muscles would become soft and weak, and he would be, in fact, an invalid.

Were he then to rise up and take moderate exercise at first, gradually increasing the amount until he had resumed his accustomed activities, his muscles would resume their former firmness and strength. Men have control over their health, and if they fail to do their part in the world it is usually their own fault. If they strive to do too much and thus impair health and shorten life, it is still their own fault. Those are happiest and live the longest who are well nourished and whose regular business demands about the same amount of bodily activity every day, as would be essential to health under any circumstances.

The object of exercise is to stimulate the vital organs to healthful activity. The breathing becomes more rapid under exercise, the blood is more rapidly oxygenized, the circulation is quickened, and the refuse matters are eliminated more promptly from the system; the digestive organs do their work more promptly and more thoroughly, and a demand is soon made for new material; the individual is hungry and takes food with increased relish. Thus the machinery of life runs on smoothly and naturally under the stimulus of fresh air and exercise, the strength is renewed and disease banished.

The best of all methods of exercise is horseback riding, and this is the reason: The horse does most of the work, and the rider receives most of the benefit, because he does not exhaust his own strength but that of the horse in taking the exercise. The rider is passive to a great extent, particularly if he has a horse with an easy gait. He is shaken up without becoming fatigued, and he really gets more air in the lungs than if he were walking. Another most important point is the excitement and pleasure of riding; it is often a new sensation, while walking is commonplace and soon becomes tiresome, and then its benefits diminish. Still walking is next to horseback riding, and is a method of locomotion that renders us quite independent.

